# Metallophosphoesterase-Domain-Containing Protein 2 (MPPED2) Expression in High-Risk Human Papilloma Virus-Induced Cervical Carcinoma and Its Correlation With p16INK4A Protein

**DOI:** 10.7759/cureus.70576

**Published:** 2024-09-30

**Authors:** T. Sujatha, E. Jayashankar, Putcha Uday Kumar, Triveni Bhopal, Reji Manjunath, Mullapudi Venkata Surekha

**Affiliations:** 1 Pathology and Microbiology Division, Indian Council of Medical Research-National Institute of Nutrition, Hyderabad, IND; 2 Pathology & Lab Medicine, All India Institute of Medical Sciences, Bhopal, Bhopal, IND; 3 Department of Pathology, Mehdi Nawaz Jung Cancer Hospital, Hyderabad, IND; 4 Medical Records Unit, Chengalpattu Government Medical College, Chengalpattu, IND

**Keywords:** cervical cancer, hpv, mpped2, p16ink4a, squamous cell carcinoma

## Abstract

Introduction

Recently, the expression of metallophosphoesterase-domain-containing protein 2 (MPPED2) was identified in cervical cancer. However, its precise role and correlation with other tumor suppressor proteins, such as p16INK4A, is not well studied in high-risk human papillomavirus (HPV) integrated human cervical carcinoma. Hence, in the present study, we try to see the expression of MPPED2 in human cervical carcinoma and its correlation with age and p16INK4A protein expression level.

Methods

The prospective study consists of 200 samples of 150 known cervical carcinoma and 50 controls. Histopathological evaluation, immunohistochemical staining, and semi-quantitative scoring of the intensity of proteins were performed. Statistical analysis was performed with the Shapiro-Wilk test, Spearman's rho correlation sig. (two-tailed), and Student's t-test.

Results

The data show that among the 150 cases, 136 (68.0%) cervical carcinoma tissues express the presence of high-risk HPV viral genome integration in the host cell. The expression of p16INK4A protein is higher in those tissues identified with high-risk HPV viral genomes. In contrast, the expression of MPPED2 protein is lesser or absent in those cervical tissues that have the higher expression of p16INK4A protein and vice versa. There is a significant correlation (p=0.000) between age and p16INK4A protein expression but not with MPPED2. A significant linear correlation (p=0.000) is found between the p16INK4A and MPPED2 proteins.

Conclusion

It may support the therapeutic application of MPPED2 protein to prevent cervical carcinoma progression in the near future.

## Introduction

Cancer of the cervix, which is caused due to persistent infection with human papillomavirus (HPV), is the second leading cause of death due to cancer in women worldwide [1]. The available data suggests that the E6 and E7 genes, among the identified HPV genes, are primarily expressed in squamous cell carcinoma and critically regulate the cell cycle. These E6 and E7 genes produce oncoproteins that bind to p53 and retinoblastoma (Rb) tumor suppressor proteins, which are involved in regulating growth [2,3]. Studies involving HPV-16 and HPV-18 gene expression in cancers of the cervix affected by HPV 16 and 18 show that CDKN2/p16INK4A of the minichromosome maintenance (MCM) gene family and TOP2A are up-regulated in cancer cervix along with the increased expression of proteins of HPV E6/E7 genes [4]. An increased expression of p16INK4A protein has been demonstrated in cervical cancers due to functional inactivation of cell cycle G1 progression by the HPV E7 protein and is widely recognized as a suitable marker for early cancer of cervix detection [5]. The metallophosphoesterase-domain-containing protein 2 (MPPED2) has been identified, which is a member of the class III phosphodiesterases (PDEs) [6]. This protein's primary function is to cleave the 3', 5'-cyclic phosphate nucleosides into 5'-phosphate nucleosides and regulate the levels of the cyclic second messengers and their rates of degradation [7]. In humans, the MPPED2 gene is located on the 11p13 region of the chromosome and between the FSHB and PAX6 genes [8]. The MPPED2 mRNA is reported to show high expression in the brain of the fetus and is positively associated with its neurodevelopment [9]. On the other hand, expression of MPPED2 was observed to be downregulated in different cancers like neuroblastoma, cancer of the cervix, squamous carcinomas of the mouth, and breast cancer [7,9,10]. Zhang et al. reported that the integration of the HPV virus into the genome of the host cells leads to a cascade of events which further lead to further progression of cervical cancer, which thus could adversely affect the genomic sites of cervical tissues associated with cancer-related genes, such as MYC and MPPED2 [10,11]. However, no studies have been reported about the correlation of p16INK4A and MPPED2 proteins in cervical carcinoma tissue in humans.

Therefore, we tried to correlate the expression levels of both p16INK4A and MPPED2 proteins in human cervical carcinoma tissues for the first time. The main aim of the study was the identification of MPPED2 protein expression and correlation it with p16INK4A protein for a future therapeutic approach in high-risk HPV-induced cervical carcinoma.

## Materials and methods

Study population and specimen collection

The present study is a part of the study which was approved by the Institutional review board of MNJ Institute of Oncology and Regional Cancer Center and National Institute of Nutrition, Hyderabad, India (Regd No. ECR/227/Inst/AP/2013/RR-16), which included 200 samples consisting of 150 cases and 50 controls [12]. The 150 cases included histopathologically diagnosed cases of cervical cancer, while the control samples were normal cervical tissues with the absence of cancer [12]. Both cancer and normal tissues were collected in normal saline and were further processed in the lab. Papanicolaou (PAP) smears positive for epithelial cell abnormalities and cervical biopsies with dysplasia and malignancy were included, while smears and tissue sections that showed cervicitis (acute, chronic, tuberculosis), polyps, metaplasia, and hyperplasia were excluded from the study. 

Histopathological analysis

All the biopsy samples were fixed overnight in 10% neutral buffered formalin solution, processed overnight in an automatic tissue processor (Shandon Excelsior ES, Davisburg, USA), and embedded in paraffin (Shandon Histocentre 3, embedding machine by Thermo electron corporation, Waltham, USA) to form tissue blocks. These formalin-fixed and paraffin-embedded (FFPE) tissue blocks were further cut into 5 μm thick sections using a tissue microtome (Leica RM 2155, Leica Biosystems Nussloch GmbH, Nußloch, Germany) followed by staining the sections with hematoxylin & eosin (H&E) stain and immunohistochemical stain. The stained histological slides were examined for histopathological diagnosis by an experienced pathologist who recorded all the morphological changes under a bright field microscope (Nikon E800, Nikon, Tokyo, Japan) [11,13].

Immunohistochemical staining and scoring by semi-quantitative method

Expression of p16INK4A and MPPED2 proteins in the tissue sections was studied in those tissues that are identified by HPV genome expression. The antibody dilution and staining procedure were carried out as per the instructions by the manufacturer (Dako, Glostrup, Denmark). The 5 micrometers thick sections were initially treated with a blocking agent and were incubated with the p16INK4A and MPPED2 primary antibodies for 20 minutes. After thorough washing, the sections were incubated with a secondary antibody and were then tagged to HRP. DAB Chromogen (Dako, Glostrup, Denmark) was used to develop the required color of acceptable intensity, and after treatment with alcohol and xylene, the slides were counterstained with hematoxylin stain. 

An expert pathologist performed the semi-quantitative immunohistochemical (IHC) scoring of sections stained with the respective antibodies. Tissue blocks from normal women were used as controls. The sections were considered IHC positive when intense and diffuse staining for the antibody was observed in the nucleus and cytoplasm of the squamous epithelial cells of the cervical epithelium. IHC grading was performed based on the intensity of the staining, where grade 0 was given for no staining; grade 1 for weak intensity of staining; grade 2 for moderate intensity of staining; and grade 3 for strong intensity of staining. Next, the percentage of tumor cells showing the IHC staining (from an undetectable level or 0% to homogeneous staining or 100%) was estimated. We then calculated the total score by adding the IHC staining intensity with the percentage of cells staining for the primary antibodies. The maximum score was 8 and the minimum score was 0. A score ranging from 0 to 2 was considered mild IHC expression, a score ranging from 3 to 5 was moderate, and a score ranging from 6 to 8 was graded as overexpression of the antibodies [14,15].

Statistical analysis

Statistical analysis was performed using SPSS Statistics for Windows, version 25.0 (IBM Corp., Armonk, NY, USA). Shapiro-Wilk test was applied to determine the normality of the data when it showed a nonparametric distribution. To find the relationship between the variables, Spearman's rho correlation sig. (two-tailed) the method was adopted. The significance of the difference between the parameters was analyzed by using the Student-t-test, and a p-value less than <0.05 was considered to denote significance between the variables.

## Results

The study included cervical tissue samples from 200 women. Among them, 150 samples were positive for cervical carcinoma and 50 were normal. Table 1 represents the age distribution pattern of the subjects.

**Table 1:** Distribution of patient's age. N: number, %: percentage.

Age group	N	%
30-40	33	22
41-50	50	33
51-60	65	44
61-70	2	1
Total	150	100

**Figure 1:**
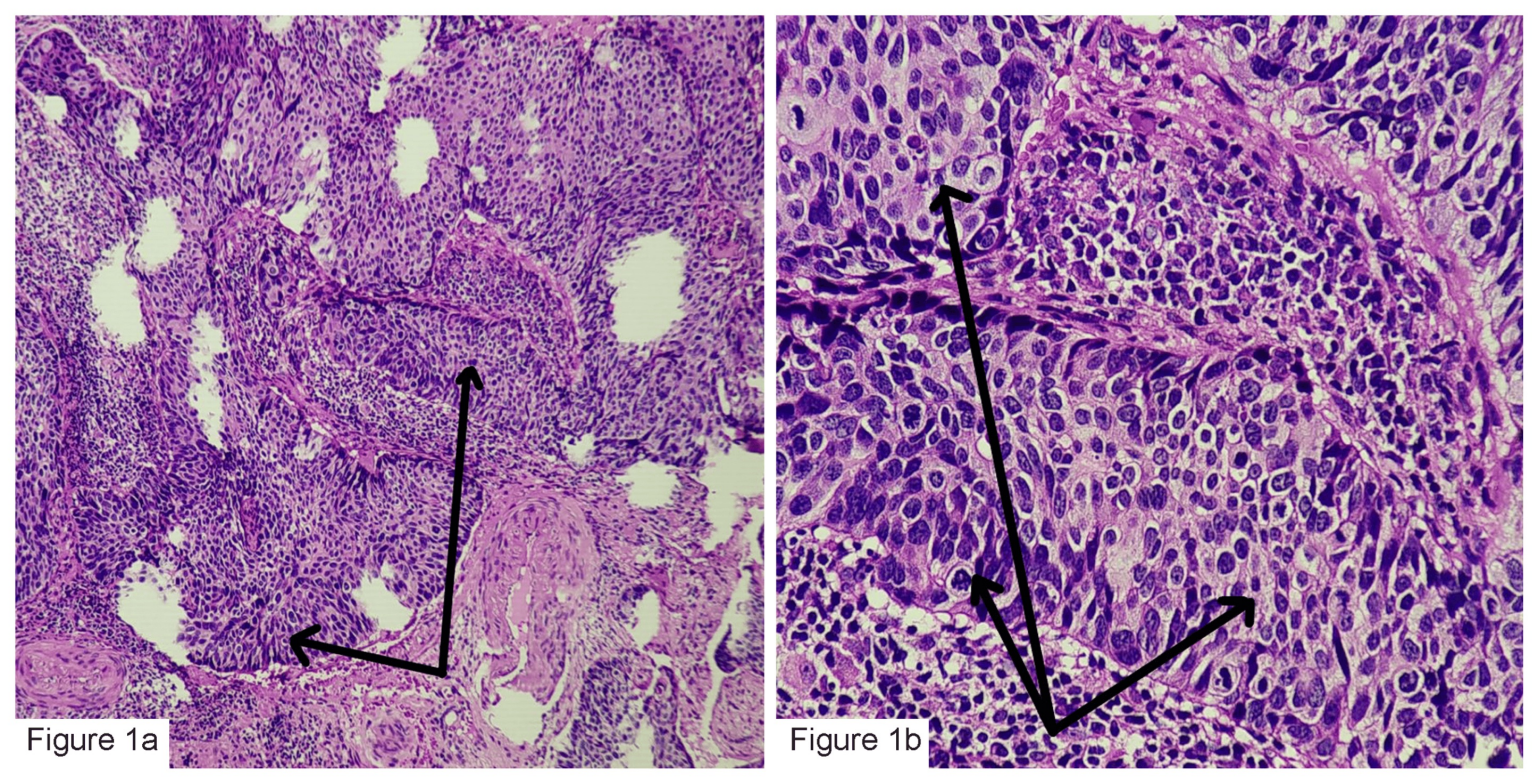
Squamous cell carcinoma of the cervix, H&E stain. (a) Squamous cell carcinoma cervix revealing features of invasive malignancy with the long black arrows pointing to malignant tumor cells, H&E (original magnification, x200). (b) Squamous cell carcinoma cervix revealing features of invasive malignancy with the long black arrows pointing to malignant tumor cells, H&E (original magnification, x400). H&E: hematoxylin & eosin.

It shows 65 patients (44%) were in the age group of 51 to 60, while only 1% (n=2) of the population belonged to the 61 to 70 age group. Hematoxylin and eosin (H&E) staining showed dysplastic features of squamous cell carcinoma (Figures 1a, 1b).

**Figure 2:**
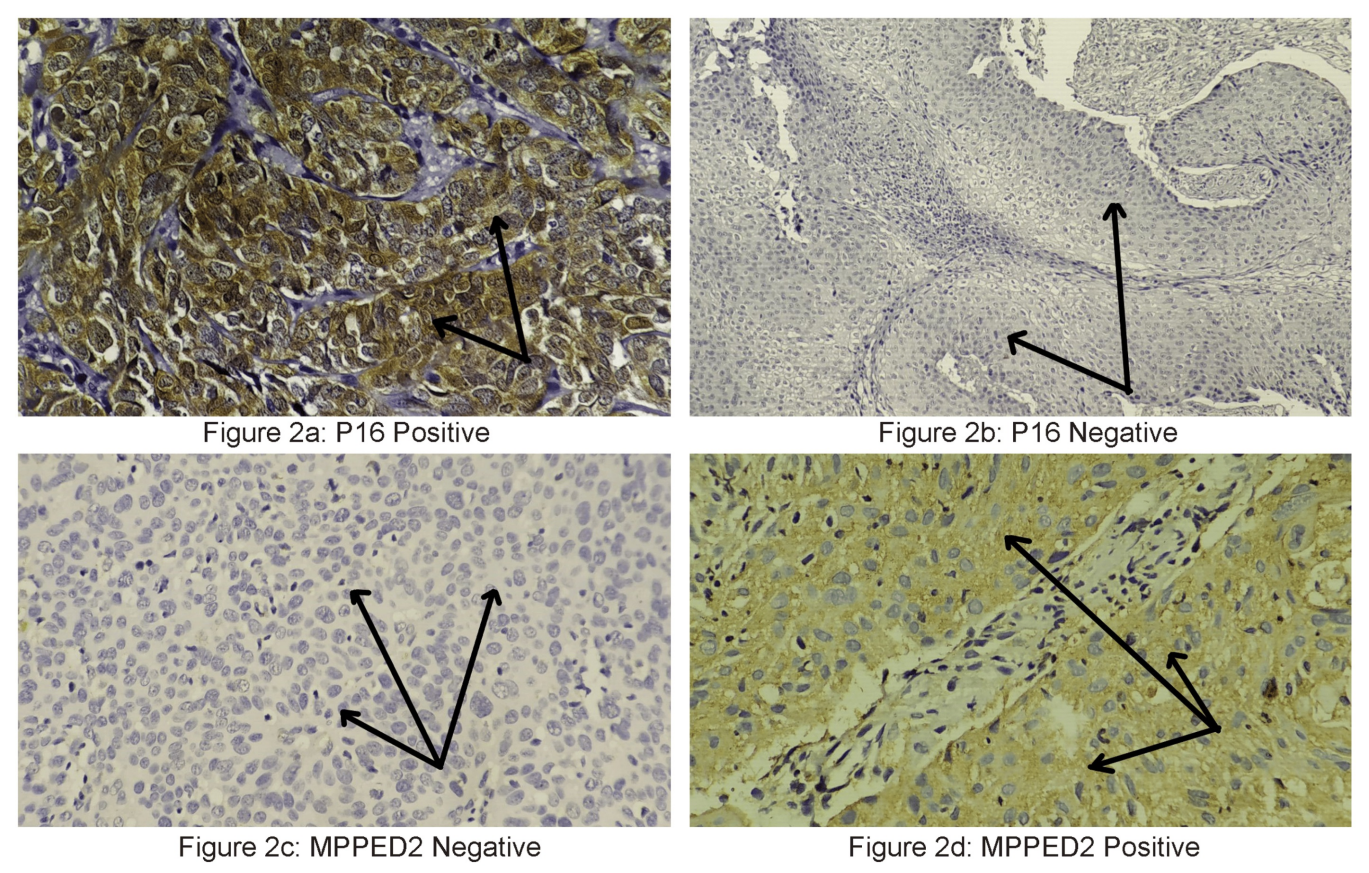
Immunohistochemical expression of p16INK4a and MPPED2 in cervical carcinoma cells. (a) Positive immunohistochemical expression of p16INK4a in cervical carcinoma cells (long black arrows) (original magnification, x400). (b) Negative or absence of immunohistochemical stain for p16INK4a in cervical carcinoma cells (long black arrows) (original magnification, x200). (c) Negative or absence of immunohistochemical stain for MPPED2 in cervical carcinoma cells (long black arrows) (original magnification, x400). (d) Positive immunohistochemical expression for MPPED2 in the cytoplasm of cervical carcinoma cells (long black arrows) (original magnification, x400). MPPED2: metallophosphoesterase-domain-containing protein 2.

Figure 2 shows the immunohistochemical expression profile of the cervical carcinoma cells for p16INK4A and MPPED2, with Figure 2a showing that the protein expression was higher in tissues with a high-risk HPV viral genome load and lower in tissues lacking the HPV genetic material (Figure 2b). The IHC staining intensity level of p16INK4A was scored and is shown in Table 2.

**Table 2:** Percentage distribution of p16 and MPPED2 IHC score rate in cancer cervix patients. IHC: immunohistochemistry, MPPED2: metallophosphoesterase-domain-containing protein 2.

IHC score rate	Frequency (p16INK4A)	Percentage (%)	Frequency (MPPED2)	Percentage (%)
0	45	30	108	72
1	4	2.6	-	-
2	-	-	5	3.3
3	6	4	12	8
4	15	10	15	10
5	5	3.4	5	3.3
6	12	8	5	3.3
7	3	2	-	-
8	60	40	-	-
Total	150	100	150	100

Sixty-three samples (42%) were grouped under the high protein expression category because their staining intensity score ranged from 6 to 8. This finding thus indicates that squamous cell carcinoma tissues with a high-risk HPV viral genome load express higher levels of p16INK4A protein. Figure 2 reveals the IHC expression of MPPED2 protein in cervical tissues. Figure 2c shows low to nil expression of MPPED2 protein in cervical tissues, showing higher expression of p16INK4A protein, while higher expression for MPPED2 is observed in tissues with low to nil staining for p16INK4A protein (Figure 2d). Table 2 shows the staining intensity score of MPPED2 in cervical tissues. The data show that IHC scoring for 108 samples (72%) ranged from 0 to 2 and was categorized into the lower expression group. Only five samples (3.3%) showed a high staining intensity score ranging from 6 to 8 and were, thus, grouped under the high-expression category.

We tried to find a correlation between age and the expression levels of p16INK4A and MPPED2 proteins. Table 3 highlights a significant correlation (p=0.000) between the age and p16INK4A protein expression level in cervical carcinoma.

**Table 3:** Correlation of age with p16INK4A and MPPED2. **Correlation is significant at the 0.01 level (two-tailed). MPPED2: metallophosphoesterase-domain-containing protein 2.

	p16INK4A	MPPED2
Age Spearman correlation sig. (two-tailed) N	0.338**	0.120
	0.000	0.091
	200	200
p16INK4A Spearman correlation sig. (two-tailed) N	-	-0.261**
	-	0.000
	-	200

On the other hand, no significant correlation is found between age and MPPED2 protein expression in cervical carcinoma. We found a significant linear correlation (p=0.000) between the expression levels of p16INK4A and MPPED2 proteins in cervical carcinoma cases.

## Discussion

Although the expression of MPPED2 protein has been identified in many cancers, no studies have been carried out to determine its significance in cervical carcinoma. Hence, for the first time, we tried to find a correlation between p16INK4A and MPPED2 protein expression in those cervical carcinoma tissues, with evidence of a high-risk HPV viral genome. The impact of HPV viral genome integration with the host cervical tissue while inducing cervical carcinoma has been well established. E6 and E7 oncogenes for viruses have been reported to be activated at the basal portion of the epithelium of cervical tissue infected by the virus [16]. In our study, expression of the E6 gene associated with the HPV viral genome with a high risk of infection of the host tissue has been observed in 136 samples (90.67%) with cervical carcinoma. It is thus considered to be the primary reason for the development of high-grade cervical carcinoma.

Research data from multiple studies have demonstrated that p16INK4A may be a valuable marker for identifying squamous and glandular epithelial dysplasia in the uterine cervix [17]. In our study, most cervical carcinoma tissues are shown to express high levels of p16INK4A, and the expression levels are also significantly found to be correlated with age and directly connected with the levels of the high-risk HPV viral genome in them. Though most of the data supported the role of p16INK4A as a surrogate marker in the early detection of cervical carcinoma, none of the research studies have tried to find out the correlation between HPV viral genome integration and p16INK4A expression with MPPED2 expression in cervical carcinoma environment. The MPPED2 protein typically regulates many essential cellular functions in mammals, including differentiation, proliferation, and apoptosis [11,18]. Down-regulation of the MPPED2 gene expression has been reported in several malignant tumors, including squamous cell carcinoma of the mouth, thyroid carcinoma (papillary variant), and cancer cervix [11,19,20]. Thus, this vivo experimental approach to identify the anti-proliferative nature of MPPED2 could be beneficially used for tumor suppression in varied cancers [18].

Our study found a linear relationship between the age and p16INK4A protein expression but not between the age and MPPED2 protein expression in cervical carcinoma patients. At the same time, an inverse correlation between the p16INK4A and MPPED2 protein levels expression in cervical carcinoma patients was also observed. To be more precise, the expression of MPPED2 protein is observed to be higher in cervical carcinoma tissues, expressing a lower level of p16INK4A protein and vice versa. We could also find a significant association between the immune level expression of both p16INK4A and MPPED2 proteins and the patient's age. For the first time, we thus report a significant correlation between the p16INK4A and MPPED2 proteins in cervical carcinoma.

Limitations

Further studies need to be carried out in a larger population with more effective research tools to highlight the future therapeutic development of MPPED2 protein against cervical carcinoma.

## Conclusions

Our work has brought to light, the association of MPPED2 protein with p16INK4A among high-risk HPV-induced cervical carcinoma. This finding may pave the way for understanding the role of MPPED2 protein in cervical carcinoma and may help in using it for early detection of and prevention of cancer progression.
